# Development and validation of MRI‐based deep learning models for prediction of microsatellite instability in rectal cancer

**DOI:** 10.1002/cam4.3957

**Published:** 2021-05-08

**Authors:** Wei Zhang, Hongkun Yin, Zixing Huang, Jian Zhao, Haoyu Zheng, Du He, Mou Li, Weixiong Tan, Song Tian, Bin Song

**Affiliations:** ^1^ Department of Radiology West China Hospital Sichuan University Chengdu China; ^2^ Department of Radiology Sichuan Provincial Corps Hospital Chinese People's Armed Police Forces Leshan China; ^3^ Institute of Advanced Research InferVision Beijing China; ^4^ Department of Pathology West China Hospital Sichuan University Chengdu China

**Keywords:** deep learning, magnetic resonance imaging, microsatellite instability, rectal cancer

## Abstract

**Background:**

Microsatellite instability (MSI) predetermines responses to adjuvant 5‐fluorouracil and immunotherapy in rectal cancer and serves as a prognostic biomarker for clinical outcomes. Our objective was to develop and validate a deep learning model that could preoperatively predict the MSI status of rectal cancer based on magnetic resonance images.

**Methods:**

This single‐center retrospective study included 491 rectal cancer patients with pathologically proven microsatellite status. Patients were randomly divided into the training/validation cohort (*n* = 395) and the testing cohort (*n* = 96). A clinical model using logistic regression was constructed to discriminate MSI status using only clinical factors. Based on a modified MobileNetV2 architecture, deep learning models were tested for the predictive ability of MSI status from magnetic resonance images, with or without integrating clinical factors.

**Results:**

The clinical model correctly classified 37.5% of MSI status in the testing cohort, with an AUC value of 0.573 (95% confidence interval [CI], 0.468 ~ 0.674). The pure imaging‐based model and the combined model correctly classified 75.0% and 85.4% of MSI status in the testing cohort, with AUC values of 0.820 (95% CI, 0.718 ~ 0.884) and 0.868 (95% CI, 0.784 ~ 0.929), respectively. Both deep learning models performed better than the clinical model (*p* < 0.05). There was no statistically significant difference between the deep learning models with or without integrating clinical factors.

**Conclusions:**

Deep learning based on high‐resolution T2‐weighted magnetic resonance images showed a good predictive performance for MSI status in rectal cancer patients. The proposed model may help to identify patients who would benefit from chemotherapy or immunotherapy and determine individualized therapeutic strategies for these patients.

## Graphical abstract

Microsatellite instability (MSI) serves as a prognostic biomarker for clinical outcomes. We developed and validated a deep learning model that could preoperatively predict the MSI status of rectal cancer based on MR and found deep learning based on MRI showed a good predictive performance for MSI status in rectal cancer patients. The proposed model may help to identify patients who would benefit from chemotherapy or immunotherapy and determine individualized therapeutic strategies for these patients.

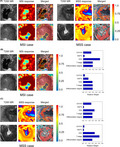

## 1 INTRODUCTION

Rectal cancer (RC) is one of the most prevalent cancers worldwide and has the second highest rate of increasing incidence among all gastrointestinal tumors.^1^ The standard treatment for locally advanced RC is surgical resection after 6‐ to 10‐week intervals of neoadjuvant chemoradiotherapy.^2^ However, the tumors show a spectrum of responses to chemoradiotherapy even within the same pathological staging, ranging from complete to poor or no response.^3^, ^4^ Heterogeneity due to different molecular pathologic features between and within tumors has been proposed to be the most likely cause of these diverse clinical outcomes.^5^ In modern RC management, there is a growing interest in the molecular profiling of tumors, as this aids clinicians both therapeutically and prognostically. Identifying predictive molecular biomarkers among RC patients could help select individuals for specific treatments and improve long‐term outcomes.

Microsatellite instability (MSI), which is the consequence of loss of one or more mismatch repair (MMR) genes, has gained considerable attention because of its significant value for RC prognosis and treatment.^6^, ^7^ Previous studies have shown that RC patients with MSI show a better prognosis than those with microsatellite stability (MSS) and obtain no benefit from 5‐fluorouracil (5‐FU)‐based adjuvant chemotherapy.^8^, ^9^ Furthermore, recent evidence demonstrated that MSI is a predictive biomarker for immunotherapy.^10^, ^11^, ^12^ On 23 May 2017, the US Food and Drug Administration (FDA) approved the immunotherapy of cancer patients with MSI.^13^ This approach was the first approved tumor treatment using a common biomarker rather than specified tumor locations in the body where the tumor originated. To develop individualized therapies and maximize the benefit to patients, MSI testing was recommended by the National Comprehensive Cancer Network (NCCN)^14^ and the European Society for Medical Oncology (ESMO)^15^ guidelines for all RC patients in 2016 and 2019, respectively. Identifying MSI by immunohistochemistry (IHC) or genetic analysis of a biopsy or surgical specimens is considered the gold standard in clinical practice. However, IHC or genetic analysis present three distinct challenges: (i) routine MSI testing using IHC or genetic analysis is not universally performed because of tedious procedures and dependence on specific equipment and reagents; (ii) the risks and potential complications of invasive sampling limit the application of these methods for the real‐time monitoring of tumor biological characteristics and pathological changes^16^; (iii) tumors are temporally and spatially heterogeneous^3^; thus, the results of MSI testing may vary depending on when and where the specimens were obtained. Therefore, developing a noninvasive, easily repeatable, and comprehensive method of preoperatively predicting microsatellite status is of great clinical significance.

Deep learning algorithms provide a new classification strategy based on artificial intelligence (AI) pattern recognition of images. A typical approach of deep learning termed convolutional neural network (CNN) has shown remarkable benefits in medicine.^17^ In the field of oncology, deep learning with CNN has been used to evaluate prognosis,^18^ noninvasively predict therapeutic responses^19^ and the *KRAS* status^20^ of RC. A recent study by Kather^21^ reported that deep learning could directly predict MSI status from histology in gastrointestinal cancer. Indeed, this study identified MSI a step ahead of IHC or genetic analyses; however, this method still relies on bioptic or surgical specimens and cannot avoid the influence of intratumor heterogeneity. The radiology field relies heavily on extracting useful information from images; thus, it is a natural area to apply deep learning to enhance its clinical utility.

Magnetic resonance imaging (MRI) is the preferred imaging modality for RC in clinical practice.^22^ To the best of our knowledge, there has not been a deep learning‐based study of a potential MRI‐based signature associated with the MSI status of RC. Therefore, this retrospective study aimed to develop and validate a deep learning model based on MR images to predict the MSI status of RC preoperatively.

## 2 METHODS

### 2.1 Patients

This single‐center retrospective study was approved by the Medical Ethics Committee of West China Hospital, and informed consent was waived due to its retrospective nature. Initially, medical records of 715 patients were retrieved who had histopathologically confirmed rectal adenocarcinoma and underwent preoperative rectal MRI examinations between January 2016 and May 2019. The exclusion criteria including (i) receiving chemoradiotherapy before MRI examination (*n* = 82), (ii) without MSI testing (*n* = 87), (iii) poor image quality to draw regions of interest (ROIs), such as obvious motion artifacts caused by intestinal peristalsis or respiration (*n* = 18), (iv) small tumors (<5 mm) or those that were hard to identify on images (n = 9), and (v) mucinous adenocarcinoma (*n* = 28). After applying these exclusion criteria, a total of 491 patients were eventually enrolled in the study. All patients underwent MRI scan, and the patients were divided into a training/validation cohort (*n* = 395) and a testing cohort (*n* = 96) following a 4:1 ratio by using an unbiased random sampling method. The detailed MRI protocol was described in the Supplementary Data.

### 2.2 Clinicopathological variables

Clinicopathological characteristics of all eligible patients, including age, sex, differentiation degree, T‐stage, percentage of Ki‐67‐positive cells (Ki67%), and levels of carcinoembryonic antigen (CEA) and carbohydrate antigen 19–9 (CA19‐9), were recorded from the Electronic Medical Record (EMR) system. In the IHC testing for MMR proteins, tumors displaying intact MMR proteins were classified as proficient mismatch repair (pMMR) and expected to be microsatellite stable (MSS), whereas those with loss of one or more MMR proteins were collectively considered as defective mismatch repair (dMMR) and presumed to be MSI.^23^

### 2.3 Tumor segmentation

MR images were retrieved from the Picture Archiving and Communication System (PACS) to a local workstation for image segmentation and analysis. A gastrointestinal radiologist, with more than 10 years of experience, manually segmented the tumor regions on each of the consecutive oblique axial T2WI images using ITK‐SNAP software (v3.6.0); a total of 4151 slices with tumor regions were manually labeled (3742 slices from MSS patients and 409 slices from MSI patients). The intestinal lumen and necrotic areas of the tumor were carefully excluded from the ROIs. To ensure accuracy, when a tumor profile was uncertain, another radiologist who has worked for 20 years and has more experience in abdominal radiological diagnosis was consulted for a final decision. Both radiologists were blinded to all clinical and pathological findings.

### 2.4 Preprocessing of MR images

The intensities of MR images were first normalized to [0, 255], and a 3D cube of 96 × 96 × 16 pixels containing the tumor region was cropped from each of the MR images. Data augmentation of the entire cropped 3D cube images including shifting, rotation, and mirroring were also performed to train our model more efficiently. There were 3160 training samples during each iteration of the fivefold cross‐validation.

### 2.5 Development of predictive models

In order to better train our models and build them more robustly, we used fivefold cross‐validation in the training/validation cohort for model development and fine‐tuning, then the testing cohort was used to evaluate the performance of different models.

### 2.6 Clinical model

Multivariate binary logistic regression classifier was used to predict MSI status based on clinical characteristics. Five variables were recorded in relation to the diagnosis as follows:

(i) Differentiation degree, a polytomous variable (well = 0, moderate = 1, poor = 2);
(ii) T stage, a polytomous variable (T1 = 0, T2 = 1, T3 = 2, T4 = 3);
(iii) Ki67%, a continuous variable;
(iv) CEA level, a continuous variable;
(v) CA19‐9 level, a continuous variable.

### 2.7 Deep learning model

**FIGURE 1:**
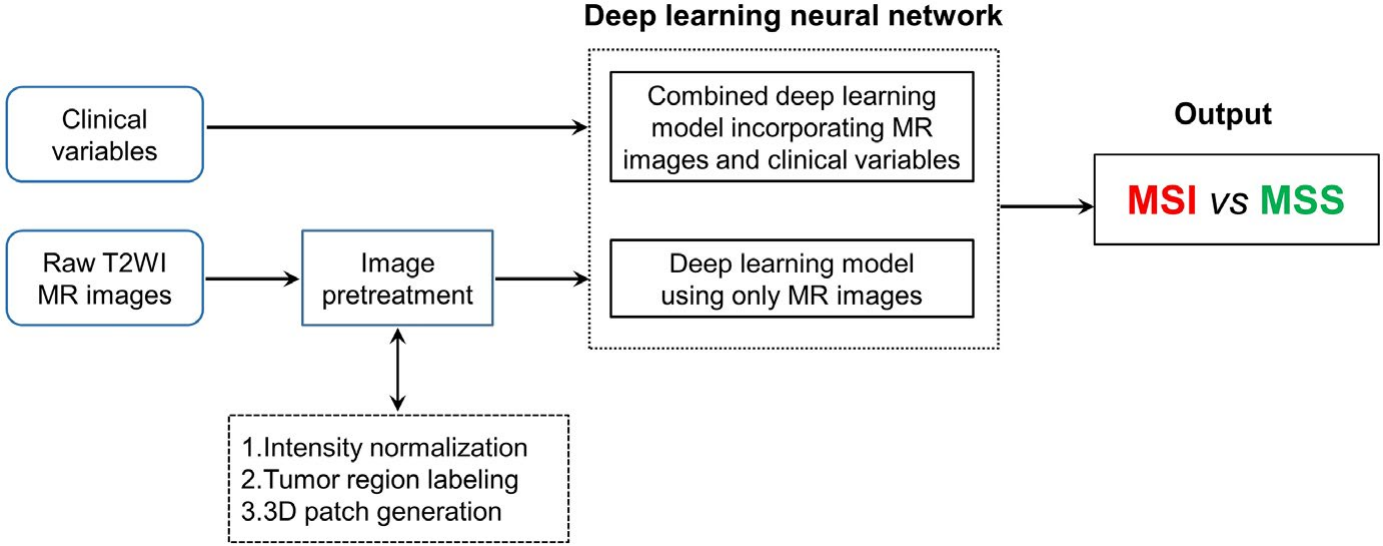
Schematic illustration of the deep learning system for microsatellite instability status prediction based on T2WI images and clinical variables. Two deep learning neural networks were designed to classify MSI and MSS in rectal cancer

Two deep learning models were designed for this study; a pure image model using only T2WI MR images and a combined model that incorporated both T2WI MR images and clinical variables. The general flow of the classification process is shown in Figure 1.

**FIGURE 2:**
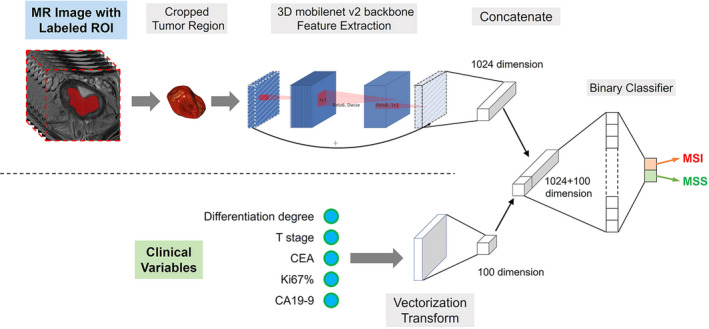
Conceptual architecture of the combined deep learning model used in this study

We adopted different architectures for the models that predicted MSI status based on imaging alone or imaging and clinical factors. For the pure image model, the 3D MobilenetV2 model with a fully connected layer was used to extract high dimension features from imaging data and to predict the probability of MSI or MSS directly. For the combined model, the clinical factors were transformed into a 100‐bit vector through a fully connected layer, which was further concatenated with the extracted 1024‐bit T2WI MR image feature. The concatenated 1124‐bit vector containing both image and clinical information was then used to predict MSI or MSS probability (Figure 2). Both weighted oversampling approach and modified binary cross entropy were used to avoid overfitting with imbalanced data. More details about the data pretreatment and model development were described in the Supplementary Data. The development and validation of the deep learning models were performed with InferScholar platform version 3.1 (InferVision).

The neural networks were implemented using Python 3.6 based on the PyTorch deep learning library and the neural networks were trained on a workstation with four GeForce GTX 1080 GPUs (NVIDIA).

The code is open source at https://github.com/zhjtwx/rectal_cancer.

### 2.8 Model explanation

To understand the most important regions in the T2WI images that contribute to the discrimination between MSS or MSI of the deep learning models, a visual explanation tool called Gradient‐weighted Class Activation Mapping (Grad‐CAM) was used.^24^ For the pure image model, we applied Grad‐CAM on the last convolutional layer of the neural networks to obtain the saliency maps, which are presented as colored heat maps to give a visual indicator of important regions on the images. For the combined model, we also showed the relative weights of each patient's clinical factors, as well as the saliency map of MR images.

### 2.9 Statistical analysis

For evaluating the capacity of the predictive models to discriminate MSI from MSS RC tumors in the training/validation and testing cohorts, a receiver operating characteristic curve was plotted and the area under the curve (AUC) was also quantified. The accuracy, sensitivity and specificity of each model was calculated based on Youden index.^25^ Delong's test was used to compare the difference between two or more AUCs of different models.^26^ The Mann–Whitney *U*‐test was used to evaluate the differences in variables with a continuous distribution across categories. The association between categorical variables was accessed by the chi‐square test or Fisher's exact test. All tests were two‐sided, and *p* values <0.05 were considered statistically significant. All analyses were performed using Prism 5 for Windows version 5.01 (GraphPad Software, Inc.,).

## 3 RESULTS

### 3.1 Study design and patient characteristics

Among the 491 patients, there were 318 men and 173 women with a median age of 61 years (range: 21–91 years). Based on the analysis of MMR proteins, the patients were classified into two groups: MSI (*n* = 51) and MSS (*n* = 440). The prevalence of MSI was 10.39% (51/491). The patients were randomly divided into training/validation cohorts (353 MSS and 42 MSI, *n* = 395) and a testing cohort (87 MSS and 9 MSI, *n* = 96). The clinical model and deep learning models were conducted in the training/validation cohort, and their performance was then assessed in the testing cohort.

There were no significant differences between the two cohorts in terms of MSI prevalence (10.63% and 9.38% in the training/validation and testing cohorts, respectively, *p* = 0.717). There were no significant differences in gender, age, tumor differentiation degree, T‐stage, Ki67%, CEA, or CA19‐9 between the MSI and MSS groups (all *p *> 0.05). Patient demographic and clinicopathological data are listed in Table 1.

**TABLE 1:** Clinicopathological characteristics of the rectal cancer patients

Characteristic	All patients (n = 491)			Training & validation cohort (n = 395)			Testing cohort (n = 96)		
	MSS (n = 440)	MSI (n = 51)	*p*	MSS (n = 353)	MSI (n = 42)	*P*	MSS (n = 87)	MSI (n = 9)	*p*
Age (years)	60.22	60.04	0.92	60.25	59.90	0.85	60.10	60.67	0.90
Sex (%)									
Male	286 (65.0)	32 (62.7)	0.75	227 (64.3)	27 (64.3)	0.99	59 (67.8)	5 (55.6)	0.46
Female	154 (35.0)	19 (37.3)		126 (35.7)	15 (35.7)		28 (32.2)	4 (44.4)	
Differentiation (%)									
G1	10 (2.3)	0 (0.0)	0.54	10 (2.8)	0 (0.0)	0.54	0 (0.0)	0 (0.0)	0.97
G2	376 (85.5)	45 (88.2)		299 (84.7)	37 (88.1)		77 (88.5)	8 (88.9)	
G3	54 (12.3)	6 (11.8)		44 (12.5)	5 (11.9)		10 (11.5)	1 (11.1)	
T stage (%)									
T1	26 (5.9)	2 (3.9)	0.59	22 (6.2)	2 (4.8)	0.32	4 (4.6)	0 (0.0)	0.82
T2	184 (41.8)	20 (39.2)		149 (42.2)	16 (38.1)		35 (40.2)	4 (44.4)	
T3	218 (49.5)	26 (51.0)		174 (49.3)	21 (50.0)		44 (50.6)	5 (55.6)	
T4	12 (2.7)	3 (5.9)		8(2.3)	3(7.1)		4(4.6)	0(0.0)	
Ki−67	58.90	54.80	0.16	59.03	55.00	0.22	58.36	53.89	0.52
CEA	16.41	12.25	0.67	16.51	13.17	0.75	16.00	7.96	0.75
CA19‐9	37.19	35.94	0.94	37.51	37.00	0.98	35.89	30.98	0.90

Differences between the two cohorts in characteristic dichotomous variables were calculated with the Chi‐squared test or Fisher's exact test, whereas the Mann‐Whitney U test was used to compare differences in actual variables.

Abbreviations: CA19‐9, carbohydrate antigen 19–9; CEA, carcinoembryonic antigen; G, grade; MSI, microsatellite instability; MSS, microsatellite stability; T, tumor.

### 3.2 Performance of the clinical model

**FIGURE 3:**
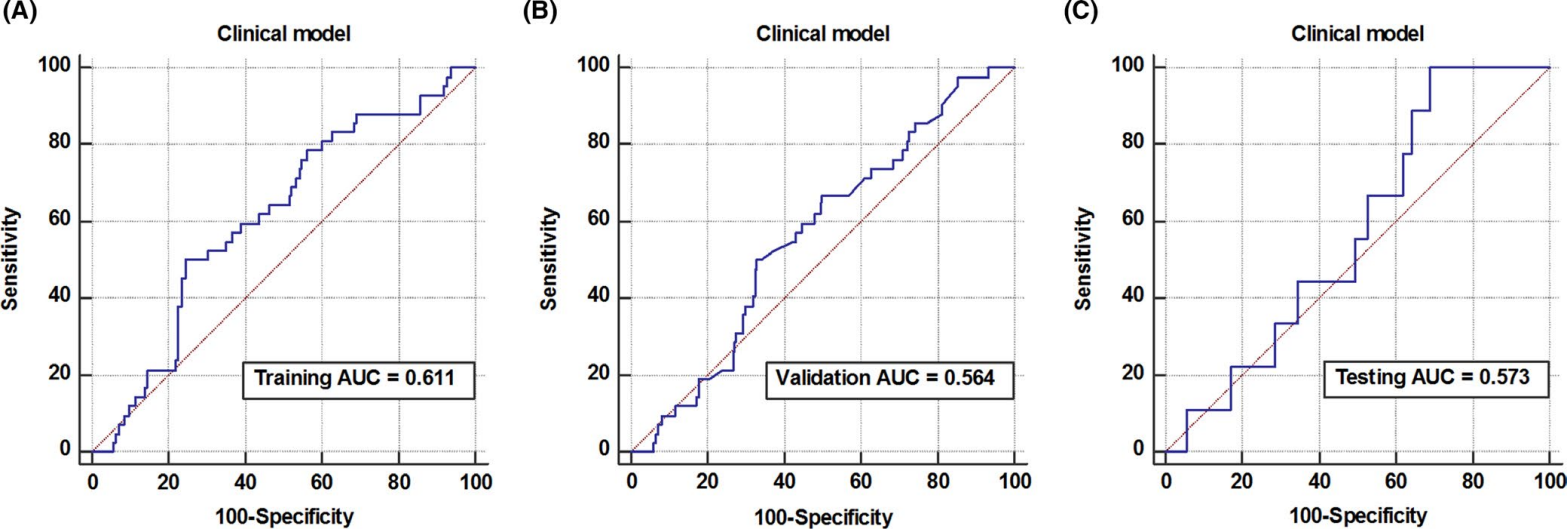
Diagnostic performance evaluation of key factor models. Receiver operating characteristic curves for the logistic regression‐based clinical model in training (A), validation (B), and testing (C) cohorts

The AUCs of the clinical model were 0.611 (95% CI, 0.561 ~ 0.660), 0.564 (95% CI, 0.514 ~ 0.614), and 0.573 (95% CI, 0.468 ~ 0.674) in the training, validation, and testing cohorts, respectively (Figure 3). The accuracy, sensitivity, and specificity in the testing cohort were 37.5%, 100.0%, and 31.0%, respectively (Threshold = 0.1328, Youden index = 0.3103). These results indicated that an effective classification of MSS and MSI in RC was not possible when only using these clinical variables.

### 3.3 Performance of the deep learning models

**FIGURE 4:**
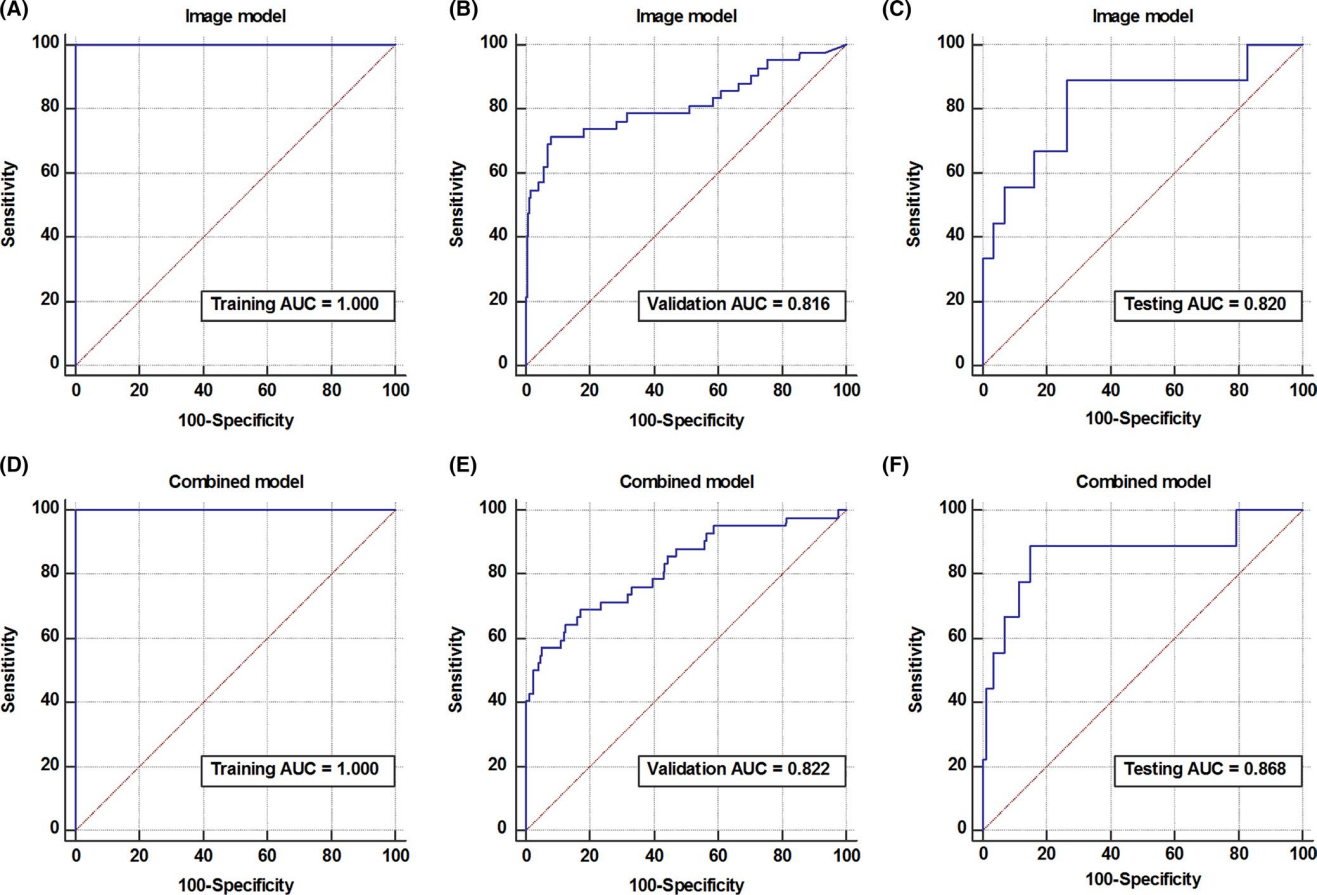
Development and validation of the deep learning models. Receiver operating characteristic curve of the pure image model in the training, validation, and testing cohorts (A–C). Receiver operating characteristic curve of the combined model in the training, validation, and testing cohorts (D–F)

The pure image model achieved AUCs of 1.000 (95% CI, 0.991 ~ 1.000) and 0.816 (95% CI, 0.774 ~ 0.853) in the training and validation cohorts. The model correctly classified 75.0% of patients regarding MSI status in the testing cohort, with a sensitivity, specificity of 88.9% and 73.6% (Threshold = 0.2727, Youden index = 0.6245), and the AUC was 0.820 (95% CI, 0.718 ~ 0.884) (Figure 4A–C). The performance of the combined model was slightly better than the pure image model, with AUCs in the training and validation cohorts of 1.000 (95% CI, 0.991 ~ 1.000) and 0.822 (95% CI, 0.791 ~ 0.859), respectively (Figure 4D–E). In the testing cohort, the combined model achieved an accuracy, sensitivity, specificity, and AUC of 85.4%, 88.9%, 85.1%, respectively (Threshold = 0.3701, Youden index = 0.7395), and the AUC was 0.868 (95% CI, 0.784 ~ 0.929) (Figure 4F). Both models showed better performance than the clinical model in the testing cohort (combined model vs. clinical model, *p* < 0.01; image model vs. clinical model, *p* = 0.04). The AUCs of the pure image model and combined model in the testing cohorts were not significantly different by Delong's test (*p* = 0.729).

### 3.4 Visualization of learned features

**FIGURE 5:**
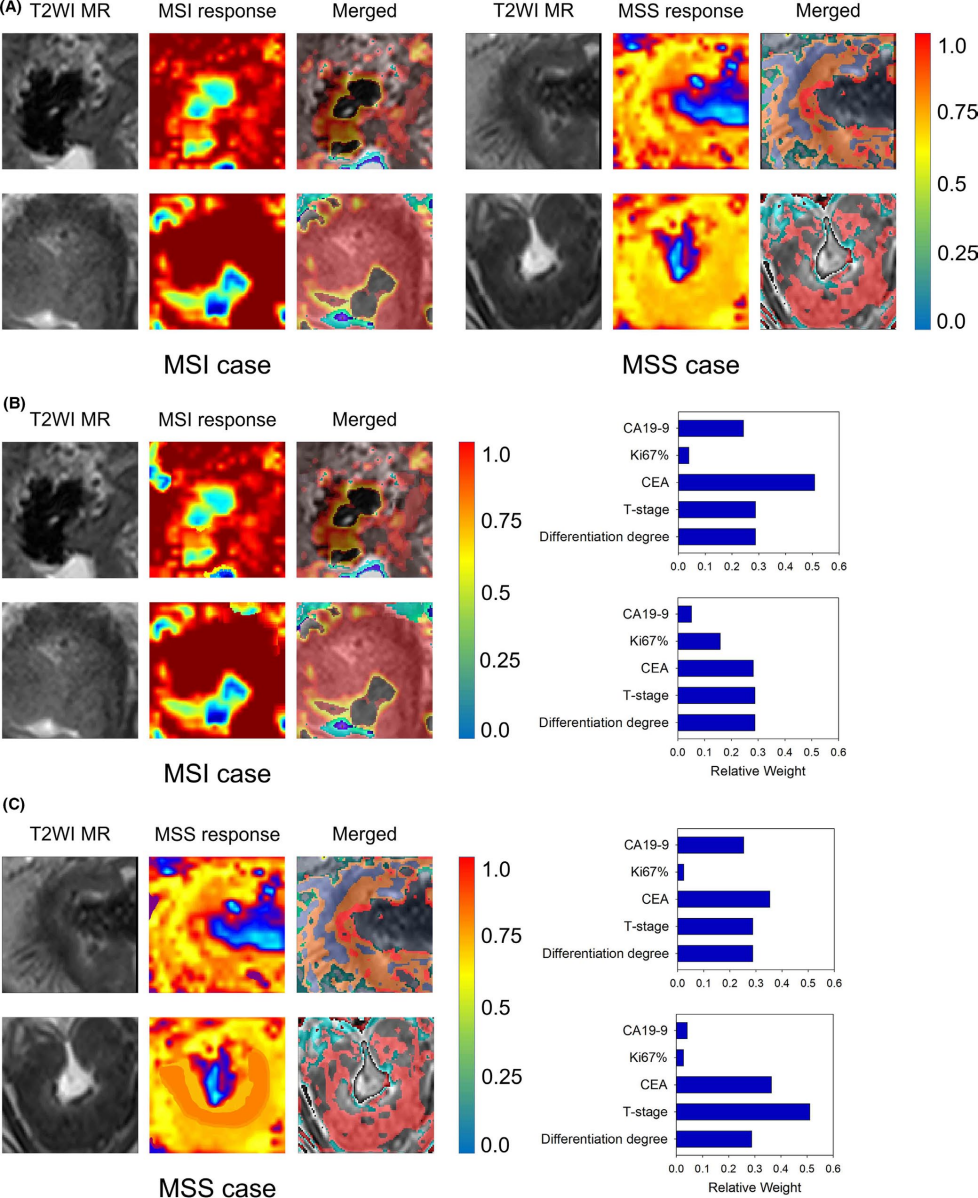
Examples of saliency map analysis. (A) The response heatmaps of the pure image model for typical MSI (left) or MSS (right) cases are presented. (B) The response heatmaps and relative weights of clinical factors of the combined model for typical MSI cases. (C) The response heatmaps and relative weights of clinical factors of the combined model for typical MSS cases. By superimposing on the input image, heatmaps highlight regions that were important in making the diagnosis for the neural network. Red indicates a stronger contribution than yellow, and blue regions had little contribution to the prediction

To examine our deep learning models and the features learned from the cohorts, we visualized the most salient regions of the T2WI MR images used by the model to make the predictions in Figure 5. According to the network structure, we adopted Grad‐CAM, a method for a visual explanation of deep networks via gradient‐based localization, to generate a class‐specific activation map on MRI slices. These saliency maps highlighted the regions of visual features that responded to the relevant prediction. The saliency maps of the same patient showed high similarity between pure image model and combined model, while the relative weights of clinical factors in the combined model were different, indicating that the contribution of MR images and clinical factors were relatively independent. The highlighted regions of saliency maps contained both tumor and peri‐tumor areas, which may be related to the fact that the MSI tumor showed a trend toward increased submucosal invasion.^27^

## 4 DISCUSSION

Our study is the first one to establish a deep learning model that predicts the MSI status of RC patients based on preoperative MR images. The combined model that integrated clinical variables and image characteristics achieved the best predictive performance with higher AUCs than the pure image model. However, the performances of the two models were not significantly different, which may be related to the failure of clinical variables to contribute significant information to the model. Therefore, an MRI‐based deep learning model alone may provide sufficient information to determine RC patients' MSI status and guide individualized treatment.

Because IHC and genetic testing are not routinely performed in many institutions, a recent study identified the MSI status of gastrointestinal cancers directly from histological sections using deep learning methods and obtained a good prediction performance.^21^ While this approach predicted MSI status a step ahead of IHC and genetic testing and was easy to implement, the acquisition of histological specimens still requires an invasive procedure and can only provide information on a small region of the tumor. In this study, deep learning, with a unique advantage in medical image analysis, was used to predict the MSI status of RC based on MRI images and obtained a desirable predictive performance. MRI was noninvasive and provided information on the entire tumor, avoiding the complications of intratumor heterogeneity.

A CT‐based radiomic model was employed to distinguish the MSI status of colorectal cancer in recent studies.^28^, ^29^ Compared with these studies, our study had three improvements. First, these prior studies included all left‐ and right‐sided colorectal cancers that exhibit significant pathological differences, including the MSI status.^30^ We focused only on RC in this study to reduce the bias caused by pathological differences in left‐ and right‐sided colorectal cancers. Secondly, compared with CT, MRI played a pivotal role in the pretreatment assessment of RC and provided higher soft‐tissue resolution,^21^ allowing tumor borders to be delineated more accurately. Thus, MRI provided more valuable data for the high‐throughput extraction of quantitative image features. Third, deep learning networks are multi‐layer feed‐forward neural networks that can be trained end‐to‐end in a supervised method while learning highly discriminative image features, eliminating the requirement of hand‐crafted radiomic features of images.^31^ Therefore, we employed the deep learning approach and established and validated a robust model for predicting MSI in RC based on MRI.

It has been reported that the prevalence of MSI in colorectal cancer is approximately 15%, with a gradual decrease in its distribution from the proximal colon to the rectum.^30^ This scenario was reflected in the low MSI prevalence of RC in our study (10.39%), resulting in far more negative (MSS patients) than positive samples (MSI patients). Classifying imbalanced data could be problematic as the classifier built from an imbalanced cohort is more likely to be biased toward the majority class and show poor performance in the minority class.^32^ Traditional data‐level methods aimed at rebalancing class distributions such as over‐sampling minority classes or down‐sampling majority classes have been applied in previous studies.^28^, ^33^ However, over‐sampling can lead to overfitting due to the repeated use of duplicated samples. Conversely, downsampling discards data in the majority class, resulting in the loss of information.^34^ In the present study, we used algorithm‐level methods to mitigate model learning bias toward majority classes by raising the importance of minority classes. By setting a higher penalty for minority class samples and using a batch‐wise minority class rectification method,^35^ we modified the deep learning neural networks giving more emphasis to the minority classes. Our cost‐sensitive learning and per‐batch balancing strategy adjusted interclass imbalance and benefited the overall model development. Nevertheless, classifying imbalanced data remains one of the most challenging problems in machine learning.

One unique strength of our study was the combination of imaging and clinical variables to achieve better diagnostic accuracy. An element‐wise summation approach is widely used in the multimodal fusion of medical images; however, it requires spatial consistency between feature maps of different modalities.^36^ To integrate T2WI images and clinical characteristics in the deep learning model, we applied a straightforward approach to concatenate features of the two modalities.^37^ Specifically, both MR images and clinical variables were treated with feature extractor, and a new concatenating layer was added to merge the features and form a high‐dimensional feature vector. Thus, the combined model could take full advantage of learning information from MR images and clinical characteristics to improve performance. Interestingly, integration of T2WI MR images and clinical factors did not lead to significant improvement of discrimination performance than that of the pure‐image model, probably because the clinical factor‐based model could hardly discriminate MSI from MSS. The saliency map analysis also showed that the response heatmap of T2WI MR images had no significant changes when combined with clinical factors, indicating that clinical factors' contribution to final prediction was quite small.

It should be noted that this preliminary study has limitations. First, although our study included 491 RC patients, which was a relatively larger sample size than previous studies^28^, ^29^ that used radiomics to predict MSI in colorectal cancer, it was still too small for deep learning, especially for a CNN with millions of weights to learn. Further data collection and studies with larger sample sizes are needed. Second, the study lacks external validation since it is a single‐center retrospective study. Thus, the reproducibility and generalizability of our prediction models remain to be verified. Therefore, further multicenter study should be conducted. Thirdly, due to the irregular morphology of rectal cancer, manual segmentation was used in this study. However, manual segmentation is time‐consuming and may be a source of observer variation. In the future, it is expected to develop accurate automatic segmentation methods for rectal cancer, which may help improve efficiency and eliminate the subjective effects of manual segmentation. Fourth, deep learning was performed only on T2‐weighted MR images in this study. Predictive performance may be improved by including other MR imaging sequences, such as diffusion‐weighted imaging and dynamic contrast‐enhanced MR imaging.

## 5 CONCLUSIONS

Our study demonstrated that deep learning based on high‐resolution T2‐weighted MR imaging had good predictive performance for RC patients' MSI status. The proposed model may help to identify patients who would benefit from chemotherapy or immunotherapy and determine individualized therapeutic strategies for these patients.

## CONFLICT OF INTEREST

The authors have declared that no competing interest exists.

## ETHICAL APPROVAL STATEMENT

This study was approved by the Medical Ethics Committee of West China Hospital (No. 2019–1159).

## Supporting information

Fig S1

Fig S2

Fig S3

Supplementary Material

## Data Availability

The data used to support the findings of this study are available from the corresponding author upon request.
