# Supplementary figures and images for: Development and validation of MRI‐based deep learning models for prediction of microsatellite instability in rectal cancer
Source: Cancer Med. 2021 May 8;10(12):4164–73. doi: 10.1002/cam4.3957 (PMC8209621; doi:10.1002/cam4.3957)

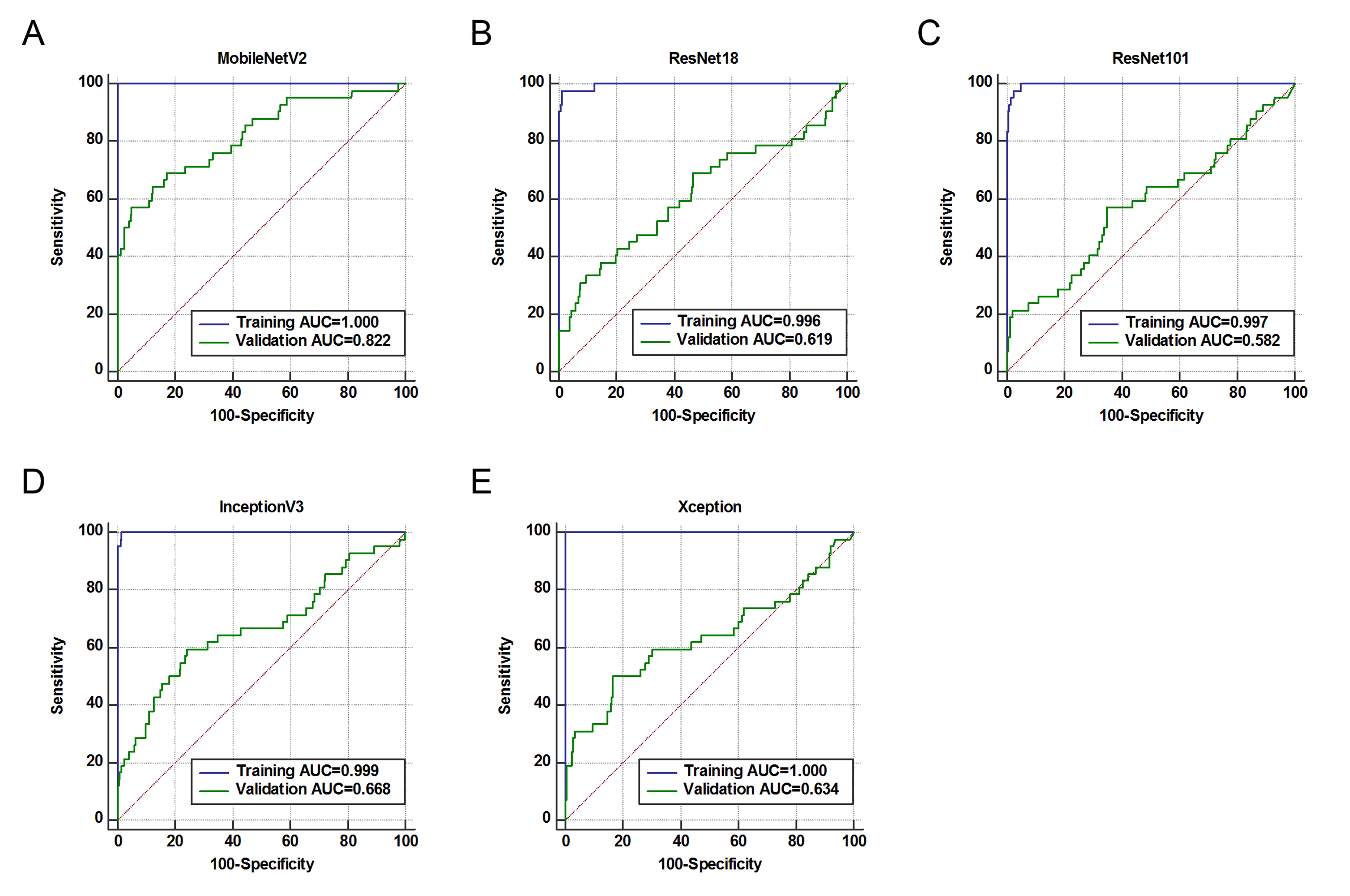

Supplement: Supplementary file 1 — Fig S1 [file CAM4-10-4164-s004.tif]

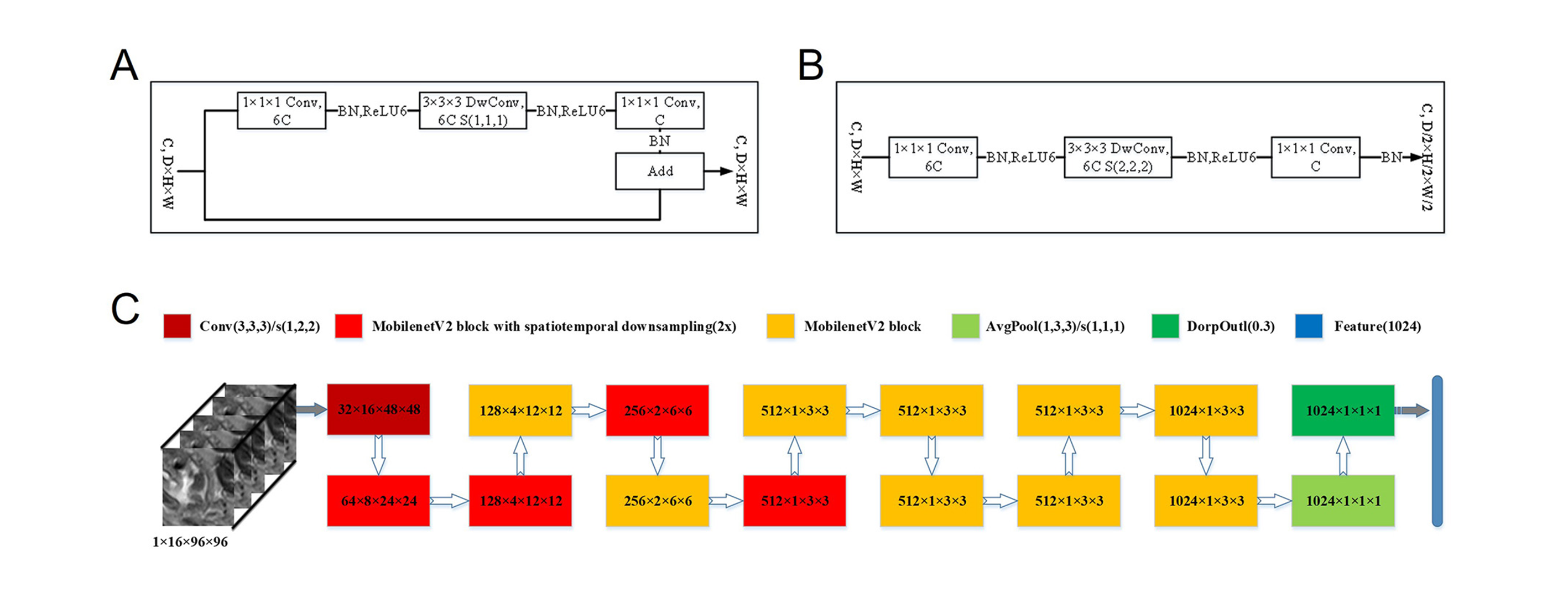

Supplement: Supplementary file 2 — Fig S2 [file CAM4-10-4164-s001.tif]

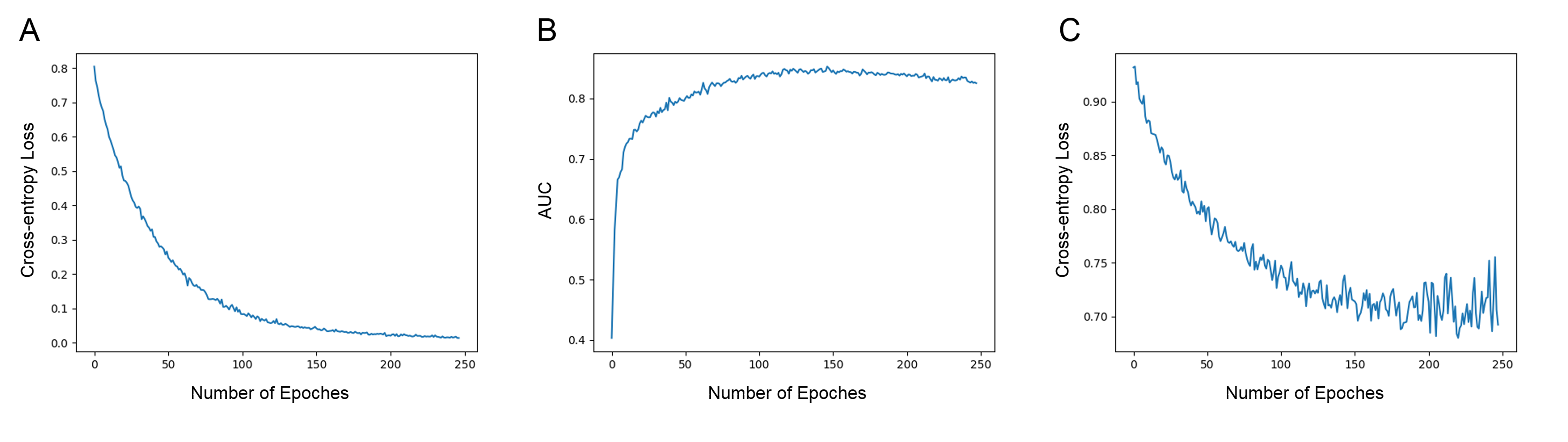

Supplement: Supplementary file 3 — Fig S3 [file CAM4-10-4164-s003.tif]
