# Supplementary material for: Development and validation of MRI‐based deep learning models for prediction of microsatellite instability in rectal cancer
Source: Cancer Med. 2021 May 8;10(12):4164–73. doi: 10.1002/cam4.3957 (PMC8209621; doi:10.1002/cam4.3957)
Supplement: Supplementary file 4 — Supplementary Material [file CAM4-10-4164-s002.docx]

**Supplementary**

**Methods:**

**MRI protocol**

All patients had undergone preoperative pelvic MRI on a 3.0-T Magnetom Skyra MR scanner (Siemens Healthcare, Erlangen, Germany) with a phased-array 18-channel body coil in the supine position. Scan protocols did not change during the 41-month study period. One hour before the MR examination, two rectal suppository pills were inserted to remove feces. Thirty minutes before MR examination, 10 mg of racanisodamine hydrochloride was injected intravenously to reduce rectal motility, unless contraindicated. Oblique axial (angulated perpendicular to the long axis of the rectal tumor) high-resolution T2-weighted turbo spin echo (TSE) images were acquired with the following parameters: TR/TE, 6890/100; slice thickness, 3 mm; voxel size, 0.3 × 0.3 × 3 mm; FOV, 180 mm; matrix, 384×346; slices, 48; averages, 3; total scanning time, 5 min and 5 s; and parallel acquisition technique with a generalized auto-calibrating partial parallel acquisition acceleration factor of 2.

**Pretreatment of MRI images**

Before developing the deep learning models, we first applied intensity normalization to rescale the intensities of the T2WI images [0, 255], the code was as follows:

def normalize(slice):

'''

input: unnormalized slice

OUTPUT: normalized clipped slice

'''

image_nonzero = slice[np.nonzero(slice)]

if np.std(slice) == 0 or np.std(image_nonzero) == 0:

return slice

else:

tmp = (slice - np.mean(image_nonzero)) / np.std(image_nonzero)

return tmp

After intensity normalization, each MRI sample was transformed and defined as follows: (i) since the deep learning models need the unified size of the input images, a 3D cube of 96 × 96 × 16 pixels containing the tumor region was cropped from each of the T2WI MR images, determined by the largest tumor region of all patients; (ii) tumor masks, in which non-lesion areas were left padded with zero, were manually labeled pixel-wise; (iii) the pathologically identified label of tumor MSI status was applied. Due to the limited amount of training data in this study, data augmentation techniques, including shifting (left, right, front, back, up, down), rotation (+30 degree, -30 degree), and mirroring, were performed for model development to train the neural networks efficiently. This yielded a total of 3160 samples for model training during each iteration of the five-fold cross-validation.

**Comparison of different neural networks**

To optimize the diagnostic performance of the deep learning model, neural networks, including MobileNetV2, ResNet18, ResNet101, Inception V3, and Xception, were used, and their performance was compared by accessing AUCs through five-fold cross-validation method in the training/validation cohort. Pre-training was widely used in the research, and application of deep learning could benefit the generalization performance of classifiers.^1, 2^ For improving the model’s adversarial robustness and transferability, all CNNs were pretrained on the natural image dataset^3^ and multiple medical image datasets from The Cancer Imaging Archive (TCIA) database^4^ in this study. The ROC curves of the combined models using these neural networks in the training and validation cohorts are shown in Supplementary Figure 1. A detailed comparison between the neural networks is summarized in Supplementary Table 1.

**Supplementary Table 1.** Comparison of adjustable parameters and diagnostic performance using MR images and clinical factors between various neural networks.

| CNN | Model Parameters | Training cohort | |  | | Validation cohort | | |
| --- | --- | --- | --- | --- | --- | --- | --- | --- |
|  |  | AUC (95% CI) | P |  | AUC (95% CI) | | P |  |
| MobileNetV2 | 3.4 Million | 1.000 (0.991~1.000) | REF |  | 0.822 (0.791~0.859) | | REF |  |
| ResNet18 | 11 Million | 0.996 (0.984~1.000) | 0.214 |  | 0.619 (0.569~0.667) | | <0.001 |  |
| ResNet101 | 44 Million | 0.997 (0.984~1.000) | 0.074 |  | 0.582 (0.532~0.631) | | <0.001 |  |
| Inception V3 | 25 Million | 0.999 (0.989~1.000) | 0.233 |  | 0.668 (0.619~0.714) | | 0.022 |  |
| Xception | 23 Million | 1.000 (0.991~1.000) | 1.000 |  | 0.634 (0.584~0.682) | | 0.003 |  |

Abbreviations: CNN, convolutional neural network; AUC, area under the curve; CI, confidence interval; REF, reference.

Overfitting was observed when the neural network’s complexity increased, suggesting that over-parameterization could lead to over-weight of measurement errors and, therefore, did not result in an accurate diagnosis.^5^ MobileNetV2 was finally used as the backbone network of the deep learning models for its high efficiency and low variance between training and validation cohorts.^6^

**Modification of the MobileNetV2 network**

Since cropped 3D patches from MRI images were used as input data in this study, the original MobilenetV2 model was modified into a 3D version according to our data characteristics; and the structure of the modified 3D block is shown in Supplementary Figure 2A-B. The architecture of the modified 3D MobilenetV2 model started with a convolutional layer, followed by four MobilenetV2 3D blocks with spatiotemporal down-sampling (2×) and nine MobilenetV2 3D blocks; a dropout layer was used to prevent overfitting (Supplementary Figure 2C), especially in cases of limited training data.

**Training of the deep learning models**

The proposed deep learning networks were trained based on the binary cross-entropy loss function, which is commonly used for classification tasks.^7^ The weights of hidden layers were randomly initialized, and the initial learning rate was set to 0.0001. The training was terminated when the loss in the validation set stopped decreasing, and the average number of epochs for the deep learning models varied from 50 to 100. An algorithm named Adam was used as the optimizer in the training stage owing to its fast convergence and weight-dependent learning rate.^8^ The plots of cross-entropy loss and test AUC in the training and validation cohorts are shown in Supplementary Figure 3.

Due to the low prevalence (~10%) of MSI in rectal cancer, the imbalance degree of class distribution was relatively high in this study. We applied two strategies to minimize the class imbalance problem during model development. First, a weighted oversampling technique was used to train the model.^9^ Briefly, only resampled mini-batches with an MSS:MSI ratio from 1:2 to 2:1 was selected for training; thus, the minority class would get more weightage depending on the severity of the unbalanced dataset. Second, a modified binary cross-entropy with MSI and MSS weight ratio of 2:3 was used as the loss function to penalize errors of the majority class and value the minority class more positively. The class weights were inversely proportional to their frequency in the training data.^10^

**Supplementary References:**

1. Hendrycks D, Lee K, Mazeika M. Using pre-training can improve model robustness and uncertainty. In: International Conference on Machine Learning. 2019.

2. Geirhos R, Michaelis C, Wichmann FA, Rubisch P, Bethge M, Brendel W. Imagenet-trained CNNs are biased towards texture; increasing shape bias improves accuracy and robustness. In: International Conference on Learning Representations. 2019. ArXiv ID: 1811.12231

3. Russakovsky O, Deng J, Su H, et al. ImageNet Large Scale Visual Recognition Challenge. *International Journal of Computer Vision*. 2015; 115:211-252. doi: 10.1007/s11263-015-0816-y

4. Clark K, Vendt B, Smith K, et al. The Cancer Imaging Archive (TCIA): Maintaining and Operating a Public Information Repository. Journal of Digital Imaging. 2013; 26:1045-1057. doi: 10.1007/s10278-013-9622-7

5. Maiwald T, Hass H, Steiert B, et al. Driving the Model to Its Limit: Profile Likelihood Based Model Reduction. *PLoS ONE*. 2016; 11: e0162366. doi: 10.1371/journal.pone.0162366

6. Sandler M, Howard A, Zhu M, Zhmoginov A, Chen L. MobileNetV2: Inverted Residuals and Linear Bottlenecks. 2018 IEEE/CVF Conference on Computer Vision and Pattern Recognition, Salt Lake City, UT. 2018, pp. 4510-4520. doi: 10.1109/CVPR.2018.00474

7. Jia Y, Shelhamer E, Donahue J, et al. Caffe: Convolutional Architecture for Fast Feature Embedding. In Proceedings of the 22nd ACM International Conference on Multimedia (MM ’14). Association for Computing Machinery, New York, NY, USA, 675–678. doi: 10.1145/2647868.2654889

8. Kingma DP, Ba JL. Adam: a method for stochastic optimization. In: International Conference on Learning Representations. 2015. ArXiv ID: 1412.6980

9. Barua S, Islam MM, Yao X, Murase, K. MWMOTE--Majority Weighted Minority Oversampling Technique for Imbalanced Data Set Learning. *IEEE Transactions on Knowledge & Data Engineering*. 2014; 26:405-425. doi: 10.1109/TKDE.2012.232

10. Yan Y, Chen M, Shyu M, Chen S. Deep Learning for Imbalanced Multimedia Data Classification. *IEEE International Symposium on Multimedia (ISM)*. 2015: 483-488. doi: 10.1109/ISM.2015.126

**Figure Legends**

**Supplementary Figure 1.** Comparison of different neural networks. The diagnostic performances of MobileNetV2 (A), ResNet18 (B), ResNet101 (C), Inception V3 (D), and Xception (E) in the training, validation, and testing cohorts are shown by ROC analysis.

**Supplementary Figure 2.** Architecture of the modified 3D block (A). A modified 3D block with spatiotemporal down-sampling (2×) (B). The modified 3D MobilenetV2 model (C). “Conv” represents the convolutional layer, “DwConv” represents the depth-wise convolutional layer, and ‘BN’ represents batch normalization.

**Supplementary Figure 3.** Plot of cross-entropy loss and accuracy during the development of the deep learning model. (A) Plot of cross-entropy loss on the training cohort. (B) Plot of diagnostic accuracy on the validation cohort. (C) Plot of cross-entropy loss on the validation cohort.
